# Peer Review of “Lessons Learned From the Resilience of Chinese Hospitals to the COVID-19 Pandemic: Scoping Review”

**DOI:** 10.2196/36347

**Published:** 2022-04-06

**Keywords:** COVID-19, pandemic, SARS-CoV-2, health care, hospitals, health care strategy, hospital resilience, interventions, crisis response, crisis preparedness, public health

*This is a peer-review report submitted for the paper “Lessons Learned From the Resilience of Chinese Hospitals to the COVID-19 Pandemic: Scoping Review.”*

## Round 1 Review

### General Comment

This paper’s [1] title mentions that the authors conducted a scoping review but used the PRISMA (Preferred Reporting Items for Systematic Reviews and Meta-Analyses) method. The authors should clarify the difference between scoping review and systematic review.

### Specific Comments

#### Major Comments

1. Provide a table of the studies that were selected for final analysis (study title, publishing year, research methods, main findings of each study)

2. State the study exclusion reasons clearly with a subheading in the Methods section

3. Revise your study limitations according to the study inclusion and exclusion criteria

4. Provide the list of all studies that were included at the initial stage without inclusion and exclusion limitations in a supplementary file

#### Minor Comments

5. English editing is required

## Abbreviations

PRISMA: Preferred Reporting Items for Systematic Reviews and Meta-Analyses
